# WHO’s first global health treaty: 10 years in force

**DOI:** 10.2471/BLT.15.154823

**Published:** 2015-04-01

**Authors:** Haik Nikogosian, Vera Luiza da Costa e Silva

**Affiliations:** a World Health Organization, Avenue Appia 20, 1211 Geneva 27, Switzerland.

It is 10 years since the World Health Organization (WHO) Framework Convention on Tobacco Control entered into force.^1^ This binding treaty, the first to be negotiated under the auspices of WHO, is widely recognized as a major milestone in global health.

Although challenges in fully implementing the treaty remain, it has been successful as a novel public health instrument. It has been ratified by 180 Parties – representing 90% of the global population – making it one of the most rapidly embraced treaties in the United Nations system.

The treaty’s legal framework has enabled the rapid adoption of key instruments to promote its implementation. There are guidelines and policy options^2^ on most substantive articles, a reporting system and most recently, a Protocol to Eliminate Illicit Trade in Tobacco Products has been adopted. All instruments have resulted from formal intergovernmental processes – an important feature underlining consensus on their technical and political strengths.

There has been a strong impact on national legislation. Of those that have reported, 80% (135/168) of Parties have either strengthened their laws or adopted new tobacco control legislation after ratifying the treaty.^3^ Accordingly, the global implementation rate of the Convention has steadily increased over the years. In 2014, the average rate of implementation of the substantive provisions of the treaty stood at almost 55%.^3^

There has been political recognition of the role of the Convention in achieving progress in key areas such as the global action against noncommunicable diseases, addressing social determinants of health and strengthening the links between the development agenda and health. This has been manifested in several high-level resolutions^4^ and declarations^5^ including at the United Nations level.^6^^,^^7^

The Protocol to Eliminate Illicit Trade in Tobacco Products^8^ complements the Convention in one of the most challenging areas of tobacco control. Illicit trade increases the accessibility and affordability of tobacco products, thus fuelling the tobacco epidemic and undermining tobacco-control policies; it also causes substantial losses in government revenues, and contributes to the funding of transnational criminal activities. The Protocol, which is a new international treaty in its own right, has demonstrated the power of the Convention to further strengthen the legal dimension of international health cooperation.

Challenges in fully implementing the treaty have also been revealed in recent years. According to Parties’ reports, the interference of the tobacco industry, including through legal challenges brought against governments, remains the most prevalent obstacle.^3^ Lack of political support and weak intersectoral coordination, as well as scarce human and financial resources are still major obstacles in some countries. While smoking prevalence has started to decline in most countries that have provided comparable data,^3^ the rising use of tobacco products that are expanding in their global reach – such as smokeless tobacco, water-pipe and electronic nicotine delivery systems – creates further challenges in fully implementing the Convention.

Another observation from the treaty era in tobacco control is the growing ambition of governments and societies to end the tobacco epidemic. This was demonstrated through strong measures such as extending smoking bans from indoor public spaces to certain outdoor areas; banning the display of tobacco products at points of sale; prohibiting the use of additives that make the products more attractive; requiring plain packages and even outlawing the sale of tobacco altogether. In a related development, several nations declared their goals, and timelines, to become tobacco-free societies.^3^

The WHO Framework Convention on Tobacco Control offers a model for addressing the negative effects of globalization on health.
